# Undergraduate Medical Research

**DOI:** 10.1590/1516-3180.2025.1431.27112024

**Published:** 2025-01-27

**Authors:** Paulo Francisco Guerreiro Cardoso, Paulo Manuel Pêgo Fernandes

**Affiliations:** I Department of Thoracic Surgery, Hospital das Clínicas (HCFMUSP), Faculdade de Medicina, Universidade de São Paulo (USP), São Paulo, SP, Brazil.; II Vice Dean, Faculdade de Medicina, Universidade de São Paulo (USP), São Paulo (SP), Brazil; Full Professor, Department of Cardiopneumology, Faculdade de Medicina, Universidade de São Paulo (USP), São Paulo (SP), Brazil; Director of the Scientific Department, Associação Paulista de Medicina (APM), São Paulo (SP), Brazil.

Following World War II, several countries started programs to advance medical, scientific, and technological development through research funding. Despite Brazil’s abundance of strategic mineral resources, the country lacked both the technology and specialized workforce required for their exploitation.

In 1946, Admiral Álvaro Alberto da Motta e Silva, a military officer and engineer representing Brazil in the newly established UN Security Council’s Atomic Energy Commission, proposed establishing a National Research Council. The National Research Council (CNPq) was founded in 1951, when then President Eurico Gaspar Dutra recognized the strategic value of science and the need to create formal programs to support and promote research in Brazil.

Álvaro Alberto referred to Law No. 1.310 of January 15, 1951, which established CNPq, as the “Golden Law of Brazilian Research.” Its purpose was to promote and stimulate scientific and technological development through research funding, researcher and technician training, partnerships with Brazilian universities, and international institutional exchanges. Since then, CNPq has carried out its broad mission, making invaluable contributions to the country’s scientific and technological progress. Its institutional mission is summarized as follows: “*CNPq aims to promote and foster scientific and technological development in the country while contributing to the formulation of national science and technology policies*.”

Currently, under the name of the National Council for Scientific and Technological Development, CNPq provides approximately 80,000 research grants across various categories and supports research in virtually all fields of study. This investment ensures Brazil’s presence in key foreign institutions while safeguarding national sovereignty through the strategic placement of Brazilian researchers in remote locations, including distant archipelagos and Antarctica.

Since their inception, Undergraduate Research Programs (UR) have provided annual grants to support undergraduate research activities. This CNPq initiative was later adopted by state research foundations across Brazil such as FAPESP, FAPERJ, and FAPERGS, as well as universities that provide funding for undergraduate students through UR programs.

CNPq’s Institutional Program for Undergraduate Research (PIBIC) was the first such program established in Brazil. Since then, PIBIC has served public and private educational and/or research institutions, with undergraduate research grants awarded directly to institutions through an open call for proposals. The institutions themselves are responsible for selecting the projects.

However, despite the steady increase in undergraduate research scholarships over the years, the CNPq acknowledges that the number of grants remains limited compared to the country’s advisory capacity and growing university enrollment.

In medicine, undergraduate research programs offer a monthly grant of R$700 for one year, with the possibility of extension at the discretion of the institution and advisor. According to the CNPq website, the program aims to spark scientific interest and nurture talent among undergraduate students through research projects under the guidance of qualified researchers.

Undergraduate researchers who are scholarship holders tend to have higher grade point averages in their degree programs. Several factors contribute to this, including exposure to a scientific research environment and interaction with researchers and advisors, who provide them with new learning opportunities based on their own experience in their research projects. This set of factors likely enhances academic performance during undergraduate studies, leading to a well-rounded education through the acquisition of specialized scientific knowledge.

Medical students participating in PIBIC programs typically aim to enhance their qualifications during undergraduate studies. The prospect of personal growth acts as a natural filter, as truly interested individuals tend to seek out programs rather than wait to be recruited by current ones.

The involvement of undergraduate researchers in clinical or experimental research projects provides numerous opportunities and new experiences. Undergraduate researchers’ interaction with their advisor and project team demystifies research, teaching them scientific investigation processes, research methodology, results interpretation, and potential clinical applications. Throughout this process, undergraduate researchers are encouraged to formally present their project methods and results through seminars, events, and scientific papers. This learning cycle ends when students complete the program by submitting an activity report and reflecting on their progress.

This experience is life-changing for medical students, often distinguishing them from their classmates, particularly when they perform well throughout the program. For high achievers, this experience substantially impacts their future choices, influencing both how they pursue opportunities and their drive for comprehensive training, ultimately helping them choose their medical specialty and career path.

Participating in undergraduate research programs during medical school considerably affects students’ academic performance. This improvement is largely due to advisors, who encourage students to actively seek information and make critical contributions to their scientific development beyond the formal curriculum. This transformative experience fundamentally shapes future career paths, helping them become more well-rounded and dedicated physicians, surgeons, researchers, and educators throughout their professional journey.

As a final note to undergraduate research supervisors, accepting this role means objectively and fully committing to shaping qualified future professionals who can become mentors, innovators, thought leaders, and knowledge disseminators for generations to come. It is, therefore, a great responsibility and a rewarding experience for advisors to contribute to and follow their PIBIC program graduates throughout their careers.
